# RETRACTION: Ubenimex Reverses MDR in Gastric Cancer Cells by Activating Caspase‐3‐Mediated Apoptosis and Suppressing the Expression of Membrane Transport Proteins

**DOI:** 10.1155/bmri/9804694

**Published:** 2026-04-17

**Authors:** BioMed Research International

RETRACTION: Q. Guo, F.‐J. Jing, H.‐J. Qu, W. Xu, B. Han, X.‐M. Xing, H.‐Y. Ji, and F.‐B. Jing, Ubenimex Reverses MDR in Gastric Cancer Cells by Activating Caspase‐3‐Mediated Apoptosis and Suppressing the Expression of Membrane Transport Proteins, *BioMed Research International,* 2019, 4390839, 14 pages, https://doi.org/10.1155/2019/4390839.

The above article, published online on 20 February 2019 in Wiley Online Library (http://onlinelibrary.wiley.com/), has been retracted by agreement between the journal Editor‐in‐Chief, Yoel Klug; and John Wiley & Sons Ltd. During the investigation of another article (Xu et al. 2018 [https://doi.org/10.1111/cpr.12515]), the publisher found that the two articles contain apparently duplicated images. Further investigation of this article found additional duplications in Figures 1, 2, 3, 4, and 5, as well as images reused in or taken from other articles by different authors: Figure 4 in Xu et al. 2018 (https://doi.org/10.1111/cpr.12515); Figure 5 in Liu et al. 2025 (https://doi.org/10.1080/15384047.2025.2500190) and Zu et al. 2018 (https://doi.org/10.1186/s13046-018-0782-7). Due to the extent of these apparent duplications, the editor has lost confidence in the results reported, and therefore the article must be retracted. The authors have been notified of this decision.

